# Long-Term Real-World Effectiveness of Aripiprazole Once-Monthly. Treatment Persistence and Its Correlates in the Italian and Spanish Clinical Practice: A Pooled Analysis

**DOI:** 10.3389/fpsyt.2022.877867

**Published:** 2022-04-28

**Authors:** José Manuel Olivares, Andrea Fagiolini

**Affiliations:** ^1^Hospital Álvaro Cunqueiro, Vigo, Spain; ^2^School of Medicine, Department of Molecular Medicine, University of Siena, Siena, Italy

**Keywords:** schizophrenia, persistence, antipsychotics, aripiprazole once-monthly (AOM), clinical practice, long-acting injectables

## Abstract

**Background:**

One of the most significant risk factors for relapse and hospitalization in schizophrenia is non-adherence to antipsychotic medications, very common in patients with schizophrenia. The aim of this analysis was to evaluate the treatment persistence to aripiprazole once-monthly (AOM) and the factors affecting it in the pooled population of two similar studies performed previously in two different European countries.

**Methods:**

Pooled analysis of two non-interventional, retrospective, patient record-based studies: DOMINO and PROSIGO. Both analyzed treatment persistence after starting AOM treatment in the real-world setting. The primary variable was persistence with AOM treatment during the first 6 months after treatment initiation. A multivariate Cox regression model was used to evaluate the influence of several baseline characteristics on the persistence.

**Results:**

The study population comprised 352 patients included in the two studies, DOMINO (*n* = 261) and PROSIGO (*n* = 91). The overall persistence with AOM treatment at the end of the 6-month observation period was 82.4%. The multivariate analysis showed that patients with “secondary school” level of education present a 67.4% lower risk of discontinuation within 6 months after AOM initiation when compared with “no/compulsory education patients” (*p* = 0.024). In addition, patients with an occupation present a 62.7% lower risk of discontinuation when compared with unemployed patients (*p* = 0.023). Regarding clinical history, patients with a Clinical Global Impression—Severity scale (CGI-S) score ≤3 present a 78.1% lower risk of discontinuation when compared with patients with a CGI-S score ≥6 (*p* = 0.044), while patients with a time since schizophrenia diagnosis ≤8.4 years present a 52.9% lower risk of discontinuation when compared with the rest of patients (*p* = 0.039).

**Conclusion:**

The AOM persistence rate observed in this study was 82.4%, which was higher than that reported in clinical trials, aligned with other real-life studies and higher than reported for other long-acting injectable antipsychotics. The persistence rate was high in complex patients, although patients with higher level of education, active occupation, lower initial CGI-S score and shorter time since the diagnosis of schizophrenia appear to be more likely to remain persistent with AOM during the 6 months after initiation.

## Introduction

Schizophrenia is a severe, chronically debilitating disorder with a course of repeating relapses in most patients (1–3). Active psychotic episodes have a detrimental effect on the course of the disorder, favoring disease progression and the occurrence of medication refractoriness, and preventing patients from regaining their previous functional and quality of life standards (4–6).

One of the most significant risk factors for relapse and hospitalization in schizophrenia is non-adherence to antipsychotic (AP) medications (7–10). Even though AP drugs AP are effective in reducing psychotic symptoms, poor treatment adherence is more the norm than the exception among patients with schizophrenia. According to some systematic studies, nearly 80% of patients are partially or fully non-adherent to oral treatments (11, 12). By contrast, long-acting injectable (LAI) APs have demonstrated an increased adherence in patients with schizophrenia, and several studies have shown that they can reduce the discontinuation, relapse and hospitalization rates (13). Recent publications have shown that the period of active psychotic symptoms after starting treatment has a significant effect on long-term functional outcomes (14). Previous research has also shown that, even after a single psychotic episode, schizophrenia has a high relapse rate (1, 15–18).

Recognizing the key factors (patient-, disease- and treatment-related) that contribute to the low rates of treatment adherence is critical in the management of schizophrenic patients. Early detection of non-adherence is also essential for their stabilization and for the prevention of psychotic relapses and, in this regard, LAI AP drugs may help with achieving adherence as early as possible, thus improving outcomes (19–21). In addition, lack of insight, efficacy/effectiveness and adverse events/tolerability issues, prior poor adherence and substance abuse were identified as other relevant contributing factors to treatment non-adherence in previous studies (22–29).

Antipsychotics have different pharmacokinetic, pharmaco dynamic, safety and tolerability profiles. Hence, in real-world clinical practice AP treatment has to be individualized, and the results of randomized clinical trials should not be the only parameter influencing the treatment decision. The evaluation of specific symptoms, disease course, medical and psychiatric histories, side effects the patient is willing to risk and, ultimately, AP effectiveness should inform the choice of AP and/or formulation for each patient. A personalized approach may therefore be the key for treatment success.

Aripiprazole once-monthly (AOM) is an atypical second-generation long-acting AP that has been shown to be effective and well tolerated in the treatment of schizophrenia (30–35). It is indicated for the maintenance treatment of schizophrenia in patients stabilized with oral aripiprazole in Europe.

Recent studies support strong evidence in relapse prevention with AOM vs. previous therapies (36–39), and a mixed-treatment comparison of randomized clinical trials found lower AOM discontinuation rates due to adverse events (AEs) relative to other long acting APs (40).

In addition, two superimposable retrospective, non-interventional, observational studies on AOM were completed: DOMINO in Italy (NCT03005769) and PROSIGO in Spain (NCT03130478). Both studies analyzed the treatment persistence after starting AOM treatment in the real-world setting.

PROSIGO evaluated the impact of patient demographics and clinical characteristics on AOM persistence (defined as the time from treatment start to discontinuation for any reason) in the first 6 months of treatment in patients that initiated AOM after being hospitalized and stabilized from an acute psychotic relapse (41).

DOMINO performed the same evaluation on a wider population of patients with a confirmed diagnosis of schizophrenia that initiated AOM in a hospital or outpatient setting (42).

The aim of the present study was to evaluate the persistence and all factors affecting it in the pooled population of two similar studies performed in two different European countries.

This was done to reach a better understanding of the effectiveness of AOM (and of its predictors) on a larger and more representative sample of patients with schizophrenia treated in a real-life clinical practice setting.

## Materials and Methods

### Data Used in the Analysis

This work analyzed the results of two non-interventional retrospective patient record-based studies:

DOMINO: A real-world effectiveness study designed to assess treatment persistence with AOM and its correlates in 261 patients with schizophrenia as per Italian clinical practice (42). Its results showed that 86% of study subjects were persistent for at least 6 months and identified a clinical profile of patients who were more likely to respond, tolerate and benefit from AOM treatment: patients with mild, moderate or relatively severe forms of schizophrenia at the time of AOM initiation [basal Clinical Global Impression (CGI) score ≤ 5, Lifetime Dimensions of Psychosis Scale (LDPS) mania score ≤ 5, and psychotic spectrum schizoid score ≤ 11]. In DOMINO, patients initiated AOM either in a hospital or in an outpatient setting.

PROSIGO*:* A non-interventional study aiming to identify the predictors of persistence in 91 patients with schizophrenia treated with AOM after an acute relapse as per Spanish clinical practice. Its results showed that 71.4% of patients were persistent with AOM treatment during the 6 months of study, and the predictive multivariate model suggested that the main factors predicting persistence with AOM treatment were fewer years since schizophrenia diagnosis and not receiving concomitant AP medications at AOM initiation time (41). In PROSIGO, patients initiated AOM after disease stabilization in a hospital setting.

### Study Populations

To be included in either study, patients had to be at least 18 years old and diagnosed with schizophrenia based on the DSM-5 criteria. Patients were excluded if they had a primary psychological condition other than schizophrenia or had taken part in a clinical trial during the retrospective follow-up period.

Patients meeting the inclusion requirements that had initiated treatment with AOM (one injection) at least 6 months prior to the inclusion visit and had given an informed consent were consecutively recruited. Informed consent signed by patients, Ethics Committee and Regulatory Authority approval were obtained according to regulations.

Retrospective data was obtained from all available source records relating to visits performed as per clinical practice (usually once monthly) from AOM treatment initiation (index date, baseline time-point) until the follow-up visit (inclusion visit).

### Study Assessments

A standardized evaluation of demographic and clinical characteristics at the index date, and the Clinical Global Impression — Severity scale (CGI-S) at the index date (43) were conducted. Safety and tolerability were also evaluated.

### Study Variables

The primary variable considered for both studies was the persistence with AOM treatment during the first 6 months after maintenance treatment initiation. Persistence was defined as the time (number of days) between the index date (AOM initiation) and discontinuation of AOM therapy. All-cause discontinuation was defined as an interval greater than 45 days between two administrations of AOM on two consecutive or on three non-consecutive occasions.

The secondary variables collected and analyzed during the two studies were:

• Gender, age, marital status, education, occupation, living situation
• Clinical history, including substance abuse and concomitant medications
• Clinical history of schizophrenia
• Time since schizophrenia diagnosis
• Health setting at treatment initiation (inpatient or outpatient setting)
• Reason to initiate treatment
• Clinical Global Impression—Severity scale (CGI-S) at index visit.

### Statistical Methods

A pooled database was developed from DOMINO and PROSIGO final study locked databases. Data management and statistical analyses were performed by a certified statistician according to a pre-specified Statistical Analysis Plan and using the Statistical Package for Social Science (SPSS version 21.0; IBM Corporation) statistical software.

Time to AOM treatment discontinuation was evaluated by means of the Kaplan–Meier method, where the event was defined as the discontinuation of AOM treatment. Patients without treatment discontinuation at the end of retrospective follow-up were considered as censored.

The primary outcome variable was the persistence with AOM treatment (yes/no) during the first 6 months after treatment initiation. A multivariate Cox regression model was used for the primary endpoint analysis. The variables with a *p* value less than 0.05 remained in the model. Estimated hazard ratios with their 95% confidence intervals were reported. A *p* value less than 0.05 was considered statistically significant. All descriptive analyses were stratified by persistence with AOM treatment and total counts. Data was summarized by means of summary statistics.

### Predictive Model

A multivariable Cox regression model was used to evaluate the influence of several baseline characteristics (independent variables) on the persistence with AOM treatment (dependent variable: time to all-cause discontinuation from AOM). According to the Protocol and the Statistical Analysis Plan all the demographic and clinical variables with a significance value <0.15 were initially included in the model.

## Results

### Demographic and Clinical Data at Baseline

The study population comprised of all the 352 patients included in the two studies, DOMINO and PROSIGO, which contributed 261 (74.1%) and 91 (25.9%) patients, respectively.

The main demographic characteristics of the studied population are summarized in Table 1. The 352 patients were predominantly male (61.1%), their mean age was 40.2 [standard deviation (SD) 12.1] years and the majority of subjects were single or divorced (83.8%). Only 21.6% of patients lived alone, while the majority (73.9%) lived with friends/family or were institutionalized. The percentage of unemployed patients was 61.6%. Regarding education, 10.9% of patients had a university degree, 30.1% had a high school diploma, 37.2% went to secondary school and 15.9% had only compulsory education or no education. Statistically significant differences between persistent and non-persistent groups were found only for the marital status and the occupational status.

**TABLE 1:** Demographic characteristics of the study population stratified by persistence.

		Persistence with AOM treatment within the first 6 months		Total (*n* = 352)
		Yes	No	
		(*n* = 290)	(*n* = 62)	
	n	290	62	352
Age (years)	Mean (SD)	39.7 (12.13)	42.4 (11.80)	40.2 (12.10)
Gender	Male	178 (61.4%)	37 (59.7%)	215 (61.1%)
	Female	112 (38.6%)	25 (40.3%)	137 (38.9%)
Marital status	Married	26 (9.0%)	10 (16.1%)	36 (10.2%)
	Living with a partner	14 (4.8%)	0 (0.0%)	14 (4.0%)
	Single	213 (73.4%)	42 (67.7%)	255 (72.4%)
	Divorced	35 (12.1%)	5 (8.1%)	40 (11.4%)
	Widow	2 (0.7%)	2 (3.2%)	4 (1.1%)
	Not available	0 (0.0%)	3 (4.8%)	3 (0.9%)
Highest level of education	No education	4 (1.4%)	4 (6.5%)	8 (2.3%)
	Compulsory education	37 (12.8%)	11 (17.7%)	48 (13.6%)
	Secondary school	113 (39.0%)	18 (29.0%)	131 (37.2%)
	High school	89 (30.7%)	17 (27.4%)	106 (30.1%)
	University degree	34 (11.7%)	4 (6.5%)	38 (10.8%)
	Not available	13 (4.5%)	8 (12.9%)	21 (6.0%)
Occupation	Paid employment	71 (24.5%)	5 (8.1%)	76 (21.6%)
	Non-paid activity	21 (7.2%)	1 (1.6%)	22 (6.3%)
	Student	12 (4.1%)	4 (6.5%)	16 (4.5%)
	Unemployed	172 (59.3%)	45 (72.6%)	217 (61.6%)
	Not available	14 (4.8%)	7 (11.3%)	21 (6.0%)
Living situation and family support	Alone	62 (21.4%)	16 (25.8%)	78 (22.2%)
	With family or friends	199 (68.6%)	41 (66.1%)	240 (68.2%)
	Psychiatric institution	19 (6.6%)	1 (1.6%)	20 (5.7%)
	Sheltered accommodation	3 (1.0%)	1 (1.6%)	4 (1.1%)
	Other	2 (0.7%)	1 (1.6%)	3 (0.9%)
	Not available	5 (1.7%)	2 (3.2%)	7 (2.0%)

Table 2 summarizes the clinical characteristics of the study population. The mean (SD) age at diagnosis was 28.6 (9.98) years and the mean (SD) time since diagnosis was 11.2 (9.84) years. The patients had experienced a mean (SD) of 2.18 (1.89) relapses within the 2 years prior to the index date and used 2.6 (1.65) AP drugs in the 5 years preceding the index date of the trial. Statistically significant difference between persistent and non-persistent patients was found for the time since diagnosis [mean: 10.5 (SD: 9.28) and 14.5 (11.62) years, respectively].

**TABLE 2:** Clinical characteristics of the study population stratified by persistence.

		Persistence with AOM treatment within the first 6 months		Total (*n* = 352)
		Yes	No	
		(*n* = 290)	(*n* = 62)	
Time (years) since schizophrenia diagnosis	*n*	288	62	350
	*n* missing	2	0	2
	Mean (SD)	10.5 (9.28)	14.5 (11.62)	11.2 (9.84)
Age at first schizophrenia episode	*n*	288	62	350
	*n* missing	2	0	2
	Mean (SD)	28.8 (9.90)	27.4 (10.35)	28.6 (9.98)
Number of previous schizophrenia relapses within the 2 years prior to the index	*n*	237	56	293
date (maintenance treatment initiation)	*n* missing	53	6	59
	Mean (SD)	1.3 (1.17)	1.3 (0.92)	1.3 (1.13)
Number of previous schizophrenia relapses within the 5 years prior to the index	*n*	215	52	267
date (maintenance treatment initiation) including the ones indicated above	*n* missing	75	10	85
	Mean (SD)	2.24 (1.99)	1.92 (1.40)	2.18 (1.89)
Number of previous AP within the 2 years prior to the index date	*n*	253	56	309
(maintenance treatment initiation)	*n* missing	37	6	43
	Mean (SD)	2.0 (1.31)	1.8 (1.24)	2.0 (1.30)
Number of previous AP within the 5 years prior to the index date	*n*	221	52	273
(maintenance treatment initiation) including the ones indicated above	*n* missing	69	10	79
	Mean (SD)	2.7 (1.68)	2.3 (1.44)	2.6 (1.65)

*AP, antipsychotic drugs.*

A history of non-adherence with AP drugs in the 3 months prior to AOM initiation was reported in 138/352 patients (39.2%), without any statistically significant differences between persistent and non-persistent cases (*p* = 0.938).

The analysis of the data collected also showed that the CGI-S evaluation at the beginning of the study included 52.5% of patients with scores between 5 and 7 (from *markedly ill* to *extremely ill*), and CGI-S of 49.6% and 66.1% in the persistent and in the non-persistent patients, respectively (*p* < 0.05).

Other psychiatric comorbidities were present in 19.6% of patients, while non-psychiatric comorbidities were found in 67.9% of the studied population. 17.2% of persistent patients showed at least one psychiatric comorbidity, compared to 30.6% of the non-persistent patients (*p* = 0.016). Regarding non-psychiatric comorbidities, 71.7% of persistent patients presented with at least one compared to 50% in the non-persistent group (*p* = 0.001). Finally, alcohol and drug use were reported in 41.9% of patients (non-significant difference between groups, 40.1% and 50% for persistent and non-persistent patients, respectively).

### Effectiveness Analysis

**FIGURE 1:**
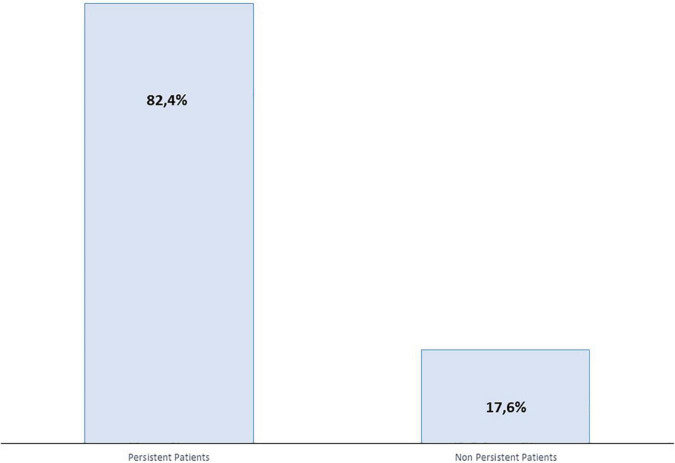
Percentage of persistent patients with AOM at the end of the 6-month analysis period.

**FIGURE 2:**
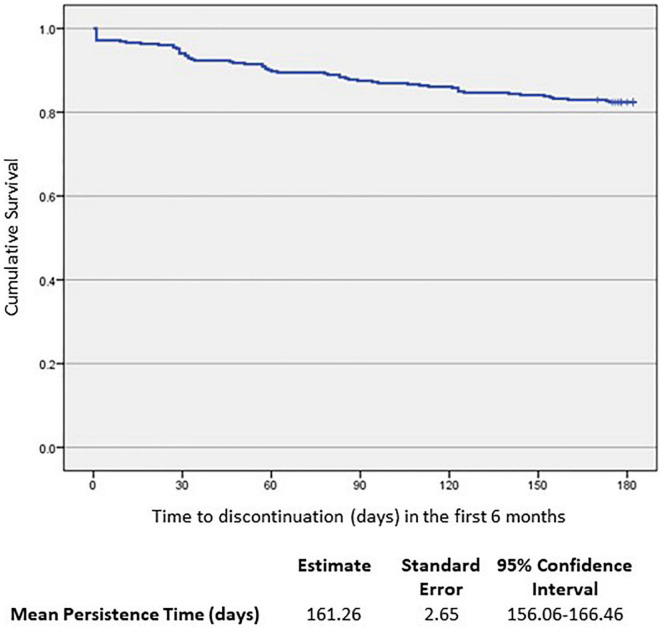
Kaplan–Meier analysis of time to AOM discontinuation (*n* = 352).

As summarized in Figure 1 and Table 3, overall persistence with AOM treatment at the end of the 6-month observation period was 82.4% (290/352 patients). Mean estimated persistence time was 161.26 days [standard error (SE) 2.65], as shown in Figure 2. In the non-persistent cohort (*n* = 62), the mean estimated persistence time was 64.24 days (SE 6.57). Mean exposure in the persistent cohort was 180 days (SE 0).

**TABLE 3:** Cumulative persistence of the study population (*n* = 352).

Cumulative persistence							
Time (days)	0	30	60	90	120	150	180
Persistent (n.)	352	330	316	308	302	295	290
Non-persistent (n.)	-	22	36	44	50	57	62

Statistically significant differences between the persistent and non-persistent groups were found only for marital status (*p* = 0.045), occupation (*p* = 0.006), time since the diagnosis of schizophrenia (*p* = 0.021, non-parametric) and CGI-S evaluation at the beginning of the study, where more severely ill patients were more likely to be non-persistent (*p* = 0.008).

### Predictive Model

After evaluating the results for association, and recategorization where appropriate, the following variables were considered for the Cox multivariate regression model:

• Age
• Time (years) since schizophrenia diagnosis
• CGI—Severity at index date
• Education
• Occupational status
• Alcohol/Drug abuse
• Concomitant schizophrenia treatments at index date
• Reason to initiate AOM: Prevent relapse/discontinuation (clinical relevance).

The final significant predictors, according to the model were: education, occupation, CGI severity at index date and time since schizophrenia diagnosis.

**FIGURE 3:**
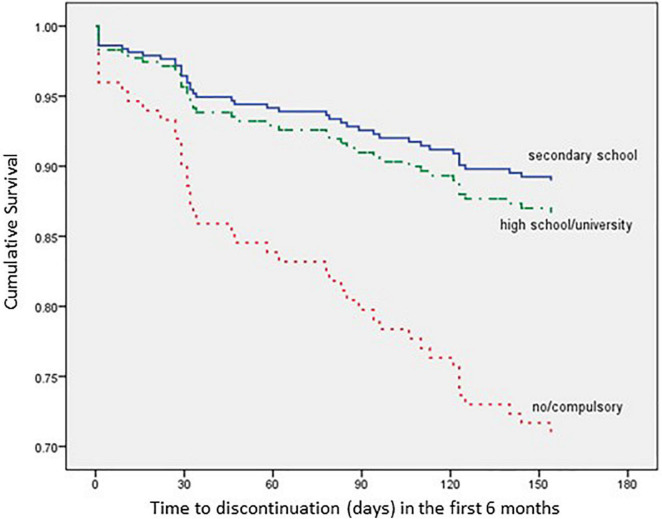
Time to all-cause treatment discontinuation in first 6 months analyzed by Education (*n* = 352).

**FIGURE 4:**
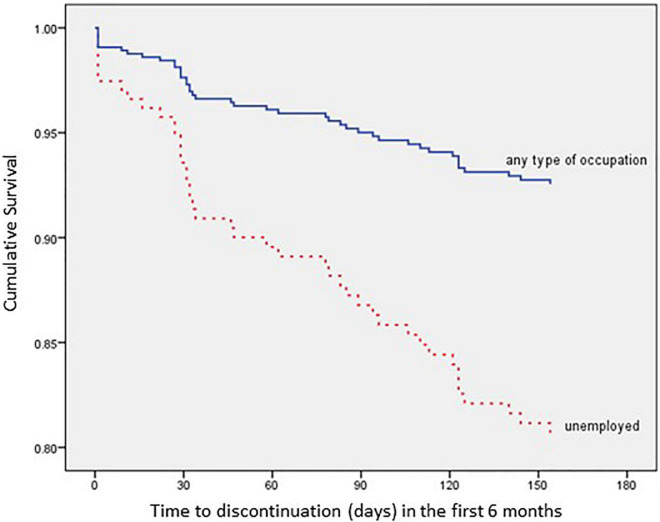
Time to all-cause treatment discontinuation in first 6 months analyzed by Occupation (*n* = 352).

**FIGURE 5:**
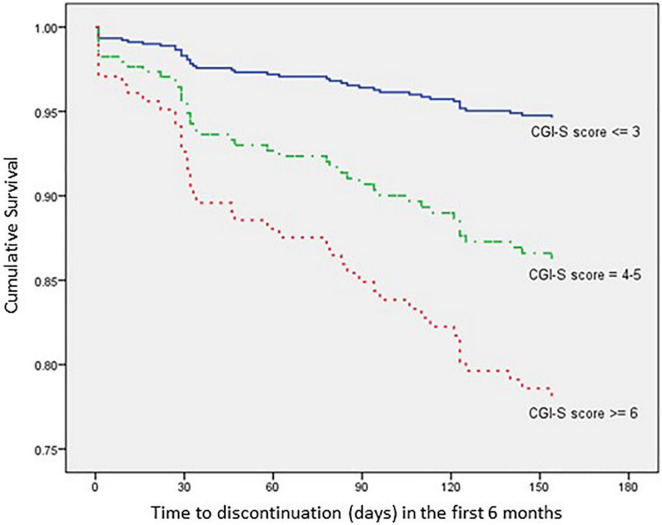
Time to all-cause treatment discontinuation in first 6 months analyzed by CGI-S score at index date (*n* = 352).

**FIGURE 6:**
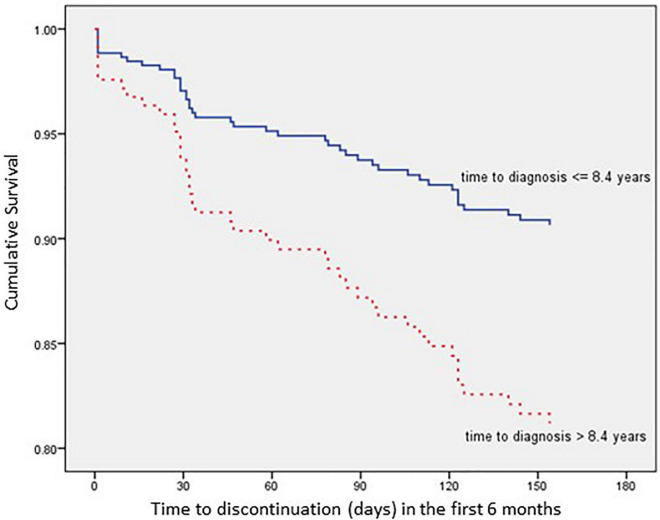
Time to all cause treatment discontinuation in first 6 months analyzed by Time since schizophrenia diagnosis (*n* = 352).

The impact of each relevant covariate, analyzed according to the Kaplan–Meier method, is detailed in Figures 3–6.

The multivariate analysis showed that patients with a secondary school level of education present a 67.4% lower risk of discontinuation within 6 months after AOM initiation when compared with no/compulsory education patients [hazard ratio (HR): 0.326; 95% confidence interval (CI): 0.141–0.753; *p* = 0.024], while patients with high school/university degree present a 60.9% lower risk of discontinuation within 6 months after AOM initiation when compared with no/compulsory education patients (HR: 0.391; 95% CI: 0.171–0.890; *p* = 0.025).

In addition, patients with an occupation present a 62.7% lower risk of discontinuation within 6 months after AOM initiation when compared with unemployed patients (HR: 0.373; 95% CI: 0.159–0.873; *p* = 0.023).

As far as clinical history is concerned, patients with a CGI-S score ≤ 3 present a 78.1% lower risk of discontinuation within 6 months after AOM initiation when compared with patients with a CGI-S score ≥ 6 (HR: 0.219; 95% CI: 0.050–0.962; *p* = 0.044), while patients with a time since schizophrenia diagnosis ≤ 8.4 years present a 52.9% lower risk of discontinuation within 6 months after AOM initiation when compared with patients with a time since schizophrenia diagnosis > 8.4 years (HR: 0.471; 95% CI: 0.230–0.963; *p* = 0.039).

As previously mentioned, only 17.6% of all patients were non-persistent. The most frequently reported reasons for discontinuation of treatment were lack of efficacy (17/352 patients, 4.8%) patient or family choice (15/352 patients, 4.3%), adherence problems (9/352 patients, 2.6%), tolerability/safety problems (6/352 patients, 1.7%).

As far as the validity of these predictors is concerned, when the outcome was treatment persistence there was no evidence of significant statistical heterogeneity between the DOMINO and PROSIGO cohorts (*p* = 0.143).

### Safety

The evaluation of the AEs and adverse drug reactions (ADRs) that occurred during the trial confirmed the well-known and overall good tolerability and safety profile of AOM.

Only 14.8% of patients (52/352) reported one or more adverse events (67 in total), while ADRs, none of which serious, occurred in 38 patients (10.8%).

Adverse drug reactions were the cause for treatment discontinuation in 26 patients (7.4%) and all ADRs with an absolute frequency ≥ 2 in the study population are listed in Table 4. No safety concerns emerged from the analysis of the pooled data.

**TABLE 4:** Adverse drug reactions occurring with an absolute frequency ≥ 2 (*n* = 352).

		Persistence	
	Total (*n* = 352)	Yes (*n* = 290)	No (*n* = 62)
Patients with at least one reaction	38 (10.8%)	17 (5.9%)	21 (33.9%)
Total number of reactions	43	20	23
**General disorders and administration site conditions**			
Patients with at least one reaction	18 (5.1%)	7 (2.4%)	11 (17.7%)
Drug ineffective	12 (3.4%)	3 (1.0%)	9 (14.5%)
Asthenia	3 (0.9%)	3 (1.0%)	0 (0.0%)
**Nervous system disorders**			
Patients with at least one reaction	16 (4.5%)	9 (3.1%)	7 (11.3%)
Akathisia	4 (1.1%)	2 (0.7%)	2 (3.2%)
Tremor	5 (1.4%)	4 (1.4%)	1 (1.6%)
Somnolence	4 (1.1%)	2 (0.7%)	2 (3.2%)

## Discussion

Treatment persistence and its predictors were assessed in this pooled analysis of two real-world, retrospective non-interventional studies (41, 42) in patients treated with AOM. The patients included in these real-world studies were from Italy and Spain, and were analyzed in two very similar studies (DOMINO and PROSIGO). In the PROSIGO study (41), only two variables at baseline were associated with the persistence of AOM; whereas in the DOMINO study (42), three variables at baseline were associated with AOM persistence (different from the variables of the PROSIGO study). To further study the predictors associated with AOM persistence, the populations were pool-analyzed to increase the sample size and robustness of the results.

The overall AOM persistence rate in the pooled population during the first 6 months of therapy was 82.4% (with a mean estimated persistence time of 161.26 days), which is higher than the 74.7–75.1% rates seen in registration trials (30, 34), in line with other real-life studies published and higher than reported for other LAI APs, such as paliperidone and risperidone (35, 51–53). In this pool analysis the percentage of AOM persistent patients increased compared to the PROSIGO study (82.4% and 71.4%, respectively), this might be explained by the baseline disease severity of PROSIGO patient population.

The observed AOM persistence can be used as an indirect indicator of the drug’s real-world effectiveness, assuming that effectiveness, tolerability and adherence are key drivers of persistence. During the study period, the safety and tolerability profile of AOM remained favorable, with few overall ADRs, none of which was serious. In fact, only 6 of the 62 non-persistent cases (9.7%) reported “tolerability problems” as the reason for treatment discontinuation. No new safety concerns were detected.

The Cox regression model showed that the patients most likely to remain persistent with AOM appear to be the ones who have a higher level of education (from secondary school to university), an active occupation, a lower initial CGI-S score (≤3) and a shorter time since the diagnosis of schizophrenia (≤8.4 years). This indicates that, the ideal patient for AOM and other APs from the point of view of persistence, is therefore an educated, occupied person, with a low CGI-S score and a short history of schizophrenia.

However, the treatment with AOM also showed good adherence rates in more complicated patients. For example, the difference in persistence in patients with a history of alcohol or drug abuse was non-significant, while still 79.2% of unemployed patients (172/217), 83.5% of single patients (213/255) and 82.4% of patients with education levels below high school (154/187) were persistent at the end of the trials. These findings suggest that AOM is an effective treatment across the whole spectrum of patients with schizophrenia.

Several investigators (especially in the past 5 years) have confirmed education (in addition to severity, occupational status and duration of condition) as a relevant predictor of treatment adherence and functioning in psychiatry (54–56). A relationship between “literacy” and adherence has also been reported in other situations where patients require chronic treatment (e.g., HIV, as reported in 57, 58).

One of the strengths of this investigation is the use of a pooled analysis, extending the study population to assess the relevance of more predictive factors and to analyze them in more detail, with a higher statistical power. Additionally, new predictors have shown to significantly influence AOM persistence (education, occupation, CGI-S and time since diagnosis) and this was not previously shown probably due to sample size limitation of the separate studies. The validity of the four above mentioned predictors was not significantly different across the DOMINO and PROSIGO populations, confirming the validity of the pooled analysis.

Regarding the study limitations, this research has the inherent drawbacks of a retrospective, non-randomized trial and it re-evaluates already published data. Additionally, there were no major differences between persistent and non-persistent patients, arguably due to a selection bias. The clinicians may have selected those patients who were more likely to respond to AOM considering several factors, including specific demographic and clinical variables associated with a higher persistence. Thus, there may not have been enough heterogeneity for those variables in the studied sample and enough power for the statistical analyses. Additionally, the set of confounding factors considered in this analysis was limited, it therefore being possible that other factors influencing the results may have been missed or overlooked.

The extended knowledge gathered through this analysis could help to increase patients’ persistence to AOM in clinical practice, and consequently the effectiveness of the drug. These results, when properly applied in clinical practice, could further improve the long-term benefits of AOM treatment in patients with schizophrenia.

## Data Availability Statement

The data analyzed in this study is subject to the following licenses/restrictions: The datasets for this article will be made available by the authors, upon request. Requests to access these datasets should be directed to JO, jose.manuel.olivares.diez@sergas.es.

## Ethics Statement

The studies involving human participants were reviewed and approved by Ethics Committee and Regulatory Authority. The patients/participants provided their written informed consent to participate in the Domino and Prosigo studies.

## Members of the Prosigo Study Group

Fernando Contreras Fernández (Hospital Universitari Bellvitge), Jordi Blanch Andreu (Numancia Salut Mental), Domenec Serrano Sarbosa (Hospital IAS Girona), Antonio Serrano Blanco (Parc Sanitari St. Joan de Deu, St. Boi), Enrique Baca García (Fundación Jiménez Díaz), Ignacio García Cabeza (Hospital General Universitario Gregorio Marañón), Javier Quintero Gutiérrez del Álamo (Hospital Infanta Leonor), Francisco Montañés Rada (Hospital Fundación Alcorcón), Carmen Jiménez Casado (Hospital Universitario Virgen del Rocío), Fermín Mayoral Cleries (Hospital Universitario Regional de Málaga), José Manuel Olivares (Hospital Álvaro Cunqueiro), Ana González-Pinto Arrillaga (Hospital Universitario de Álava), Mario Páramo Fernández (Complejo Hospitalario Universitario de Santiago de Compostela), Javier Min (Complejo Asistencial Universitario de León), and Estefanía Segura Escobar (Hospital Ciudad Real).

## Members of the Domino Study Group

AF (University of Siena, Italy), E. Aguglia (University of Catania, Italy), A. Ballerini (U Sod di Psichiatria, AOU Careggi Firenze, Italy), G. Callista (UOSD S.P.D.C. P.O. Giulianova Asl Teramo, Italy), B. Carpiniello (University of Cagliari, Italy), M. Clerici (University of Milano Bicocca, Italy), G. Corrivetti (ASL Salerno, EBRIS foundation, Italy), P. De Fazio (University Magna Graecia, Catanzaro, Italy), S. De Filippis (Neuropsychiatric clinic villa von Siebenthal-Rome, Italy), S. De Giorgi (Department of Mental Health, ASL Lecce, Italy), G. Favaretto (Department of Mental Health, Ulss2 Marca Trevigiana, Italy), E. Ferri (ASL Roma 6, Rome, Italy), G. Gargiulo (Area Vasta2, Ancona-A.S.U.R. Marche, Italy), M.G. Giustra (Otsuka Pharmaceuticals Italy), D. La Barbera (University of Palermo, Italy), G. Maina (University of Torino, Italy), C. Mencacci (DSMD—Neuroscienze Asst Fatebenefratelli- Sacco, Milano, Italy), G. Montagnani (Lundbeck Italy), A. Panariello (ASST G.O.M. Niguard, Milano, Italy), G. Pigato (University of Padova Medical Center, Italy), A. Tortorella (University of Perugia, Italy), L. Vernacotola (Otsuka Pharmaceuticals Italy), and A. Vita (University of Brescia, Italy).

## Author Contributions

Both authors contributed to the concept and design of the study, interpretation of the data, revised, and approved the final content of the manuscript.

## Conflict of Interest

The authors declare that the research was conducted in the absence of any commercial or financial relationships that could be construed as a potential conflict of interest.

## Publisher’s Note

All claims expressed in this article are solely those of the authors and do not necessarily represent those of their affiliated organizations, or those of the publisher, the editors and the reviewers. Any product that may be evaluated in this article, or claim that may be made by its manufacturer, is not guaranteed or endorsed by the publisher.
